# Concentrations of Atmospheric Culturable Bioaerosols at Mountain and Seashore Sites

**DOI:** 10.3390/ijerph16224323

**Published:** 2019-11-06

**Authors:** Byung Uk Lee, Gunwoong Lee, Ki Joon Heo, Jaeho Jung

**Affiliations:** Aerosol and Bioengineering Laboratory, Konkuk University, 120 Neungdong-ro, Gwangjin-gu, Seoul 05029, Korea; dlrjsdnd4378@naver.com (G.L.); rlwns@konkuk.ac.kr (K.J.H.); jeho0420@gmail.com (J.J.)

**Keywords:** mountain, seashore, culturable atmospheric bioaerosol, fungal bioaerosol, bacterial bioaerosol

## Abstract

Concentrations of atmospheric culturable bioaerosols at mountain and seashore sites were measured in field investigations by using a bio-culture sampler. The eastern Korean peninsula was selected for the measurements because of the short distance between the mountain site and the seashore site. Detectable concentrations of culturable fungal and bacterial bioaerosols (maximum 1065 CFU/m^3^) were quantitatively measured at the mountain and seashore sites. In addition, measurement of bioaerosols was conducted at an urban area as reference data. Significantly higher concentrations of bioaerosols were found at the mountain site. However, several fold smaller levels of bioaerosols were detected at the seashore site (*t*-test *p*-value < 0.05).

## 1. Introduction

The air in the mountains and at the seashore is generally believed to be clean because of the lack of hazardous pollutants. Residents of urban areas frequently visit the mountains and the seashore to breathe clean air in natural environments. However, we hypothesized that significant amounts of airborne microorganisms may be present in these areas. In the mountains many living organisms may release airborne microorganisms into the surrounding environments and the air at the seashore may contain many airborne microorganisms because of humid air conditions. We tried to check our hypothesis by field experiments, providing practical bioaerosol information of mountains and seashore. 

Atmospheric airborne microorganisms, known as bioaerosols, have been found to be associated with many diseases, such as pneumonia, tuberculosis, allergic rhinitis, asthma, and brucellosis [1,2,3,4,5,6,7]. Because bioaerosols are associated with various health issues, their concentrations need to be monitored to predict and prevent related human diseases. Despite their importance from a public health perspective, quantitative measurements of the concentrations of bioaerosols in outdoor air environments such as mountain and seashore locations are currently insufficient [8,9]. In this study, we quantitatively measured culturable fungal and bacterial bioaerosols at mountains and seashore sites, simultaneously. The experimental results showed that significant amounts of bioaerosols were present at the mountain site; however, several fold smaller levels of bioaerosols were detected at the seashore site. 

## 2. Experiments

**Figure 1:**
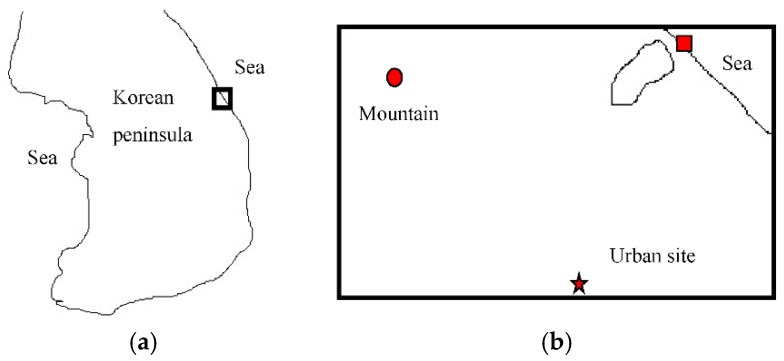
Sampling locations on the eastern Korean peninsula: mountain site (circle), seashore site (square), and urban site (star). (**a**) Korean peninsula, (**b**) Sampling locations.

Measurement of bioaerosols was conducted at a mountain site, a seashore site, and an urban area (Figure 1). All of these sites were located on the eastern Korean peninsula. The mountain site in Figure 1 was located in the foothills of one of the largest mountains on the Korean peninsula, Odae Mountain, which rises to 1565 m. The altitude of the sampling location at the mountain site was around 100 m. The seashore site was 5 km from the mountain site. The seashore site was the heavily used beach Kyung Po, which is visited by about one million people in mid-summer. However, it was nearly empty during the sampling events from February to April, because of low temperatures during spring in this area. The large bus terminal in Gangneung City (population ~ 200,000 except in the summer holiday season) was selected as the urban sampling location, as hundreds of buses and thousands of passengers use this terminal every day. 

We measured outdoor fungal and bacterial bioaerosols from February to April, during mid spring on the Korean peninsula. The sampler (Bio-culture sampler, Buck bio-culture, Model B30120, A.P. Buck, Inc., Orlando, FL, USA), which has been used in previous bioaerosol monitoring studies [10,11,12], was used to sample fungal and bacterial bioaerosols. This sampler is an impaction type sampler with a flow rate of 100 L per minute. Fungal and bacterial particles entrained in the air flow were accelerated through the 400 nozzles of the sampler and the fungal and bacterial particles were diverted from the stream and deposited onto the agar plate of the sampler. We sampled bioaerosols for 2 min and 5 min in consideration of bioaerosol concentrations (three samples per each measurement experiment). We incubated the potential fungal particles on agar containing MEA (maltose 12.75%, dextrin 2.75%, glycerol 2.35% peptone 0.75% and agar 15%) at 25 °C for 48 h. Potential bacterial particles were deposited onto the agar plate containing nutrient components (beef extract 3%, peptone 5%, and agar 15%). We incubated the bacterial particles at 37 °C for 24 h. We enumerated the number of colonies and calculated the concentrations of culturable fungal and bacterial bioaerosols in CFU/m^3^ with positive-hole corrections [10,11,13,14]. 

In addition to measurement of bioaerosols, we measured concentrations of aerosol particles by using an optical particle counter (Portable Particle Counter, Model 3905, Kanomax, Andover Township, NJ, USA) to investigate any relevance of aerosol particles to culturable bioaerosol concentrations [15]. At least three replicates were sampled and enumerated at all conditions. 

## 3. Results and Discussion

We conducted measurements of fungal and bacterial bioaerosols for three months. Table 1 shows one set of typical experimental results for April. For fungal bioaerosols, the concentration at the mountain site was 805 ± 344 CFU/m^3^. However, the concentration was 10.7-fold lower at the seashore site (75 ± 13 CFU/m^3^) on the same day (*t*-test *p*-value < 0.05). For bacterial bioaerosols, the concentrations were 775 ± 361 and 150 ± 40 CFU/m^3^ at the mountain and seashore sites (*t*-test *p*-value < 0.05), respectively. The concentrations at the bus terminal (urban site) were between those of the mountain and seashore sites. Differences in temperature and relative humidity among the sites was not significant enough to produce large differences in these concentrations [16]. 

Table 2 shows another set of experimental results for February. The average concentrations of fungal and bacterial bioaerosols at the seashore site were 4-fold (*t*-test *p*-value > 0.05) and 7-fold (*t*-test *p*-value < 0.05) smaller, respectively, than those at the mountain site. 

**Figure 2:**
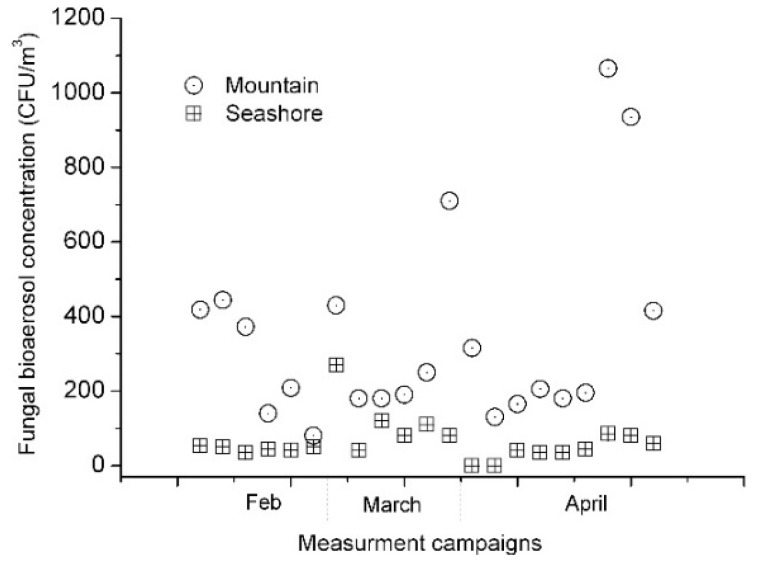
Concentrations of culturable fungal bioaerosol particles: mountain site (circle) and seashore site (square).

**Figure 3:**
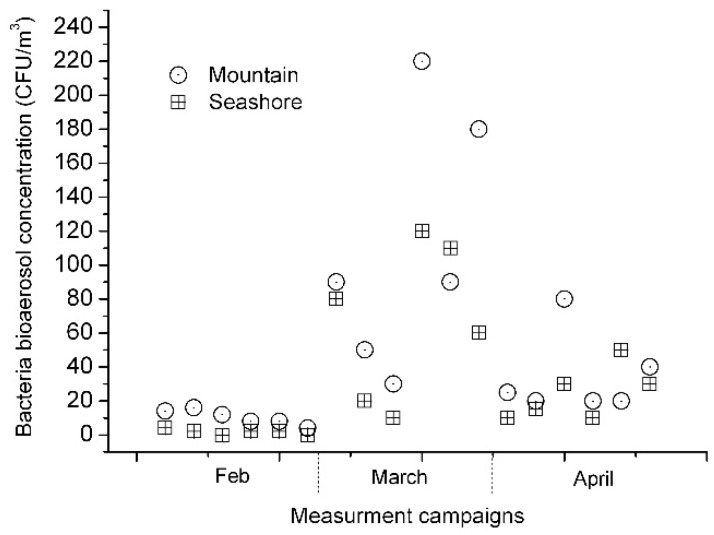
Concentrations of culturable bacterial bioaerosol particles: mountain site (circle) and seashore site (square).

Figure 2 and Figure 3 show our experimental results during three months. Measurement experiments were conducted on 24 February, 25 February, 21 March, 22 March, 6 April, 12 April, and 26 April. They show similar trends, with significant amounts of fungal and bacterial bioaerosols at the mountain site and several fold smaller amounts of bioaerosols at the seashore site. The concentrations of fungal bioaerosols at the mountain site ranged from 130 CFU/m^3^ to 1065 CFU/m^3^, while those at the seashore site ranged from 0 CFU/m^3^ to 270 CFU/m^3^. The concentrations of bacterial bioaerosols at the mountain site ranged from 4 CFU/m^3^ to 1064 CFU/m^3^, while those at the seashore site ranged from 0 CFU/m^3^ to 195 CFU/m^3^. 

**Figure 4:**
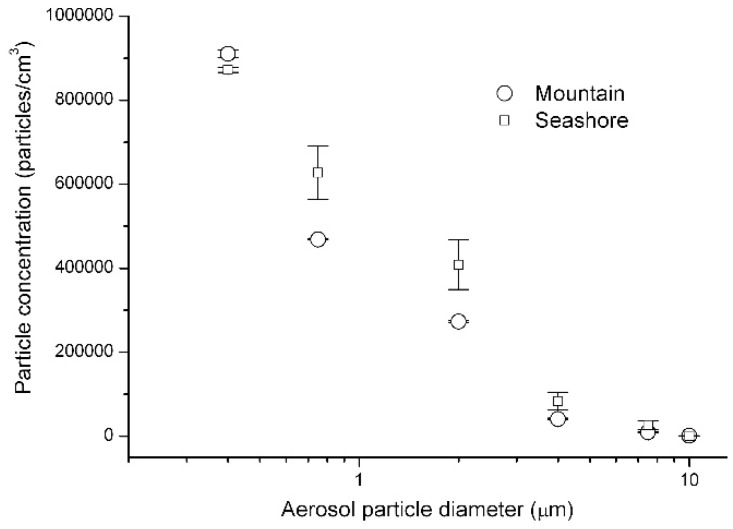
Concentrations of aerosol particles: mountain site (circle) and seashore site (square).

Figure 4 and Table 3 show the concentrations of aerosol particles at the mountain site and the seashore site. Except for very tiny particles (<0.5 µm), particle concentrations at the seashore site were higher than the concentrations at the mountain site. In particular, for fine particles (particle diameter ranging from 0.5 µm to 5 µm; typical sizes of bioaerosol particles), there were 25% to 50% more aerosol particles at the seashore site than at the mountain site (*t*-test *p*-value < 0.05). However, for culturable fungal bioaerosols, the concentration at the mountain site was 411 ± 36 CFU/m^3^ and the concentration was 9-fold lower (46 ± 10 CFU/m^3^) at the seashore site on the same day (*t*-test *p*-value < 0.05), as shown in Table 3. For bacterial bioaerosols, the concentrations were 14 ± 2 and 2 ± 2 CFU/m^3^ at the mountain and seashore sites (*t*-test *p*-value < 0.05), respectively. Therefore, the overall aerosol particle concentration was not main reason for the difference of bioaerosol concentrations between the mountain site and the seashore site. 

There could be several explanations for these findings of bioaerosol concentrations at the mountain and seashore sites. Trees and other living organisms in the mountains could produce fungal and bacterial bioaerosols [17]; therefore, the concentrations of bioaerosols in the mountains could be higher than those at urban and seashore sites. The humid environment at the seashore is adequate to support bioaerosols. However, airborne sea salt particles in this area may inhibit survival of culturable bioaerosols in the air environments [18]. More studies based on laboratory-scale investigations for the mechanisms of variations in bioaerosol concentrations are necessary to elucidate the current experimental findings. In addition, in this study, we measured culturable bioaerosol concentrations in limited periods; therefore, future measurements in extensive seasons are necessary to provide more comprehensive conclusions. Potential parameters such as wind velocities, wind directions, and microscopic identifications of microorganisms can be tested in future studies. 

## 4. Conclusions

Culturable bioaerosol concentrations were measured in mountain, seashore, and urban areas on the Korean peninsula. Significantly higher levels of bioaerosols were found in the air at the mountain site, while several fold smaller amounts of bioaerosols were detected at the seashore site in the field experiments. Further mechanism studies are necessary to investigate the reasons for this phenomenon. 

## Figures and Tables

**Table 1:** Concentrations of culturable bioaerosol particles (April).

Location	Fungal Bioaerosol Concentration (Temperature, Relative Humidity)	Bacterial Bioaerosol Concentration (Temperature, Relative Humidity)
Mountain	805 ± 344 CFU/m^3^	775 ± 361 CFU/m^3^
	24 °C, 29%	24 °C, 29%
Seashore	75 ± 13 CFU/m^3^	150 ± 40 CFU/m^3^
	26 °C, 20%	28 °C, 18%
Urban area	102 ± 11 CFU/m^3^	232 ± 133 CFU/m^3^
	30 °C, 15%	29 °C, 18%

**Table 2:** Concentrations of culturable bioaerosol particles (February).

Location	Fungal Bioaerosol Concentration (Temperature, Relative Humidity)	Bacterial Bioaerosol Concentration (Temperature, Relative Humidity)
Mountain	907 ± 825 CFU/m^3^	70 ± 10 CFU/m^3^
	12 °C, 30%	12 °C, 30%
Seashore	230 ± 53 CFU/m^3^	10 ± 10 CFU/m^3^
	8 °C, 48%	8 °C, 48%
Urban area	437 ± 131 CFU/m^3^	20 ± 10 CFU/m^3^
	10 °C, 25%	10 °C, 25%

**Table 3:** Concentrations of aerosol particles and culturable bioaerosols (d_p_ = particle diameter).

Particles	Mountain	Seashore
0.5 µm ≤ d_p_ < 1.0 µm	4.7 × 10^5^ ± 1.4 × 10^3^ particles/m^3^	6.3 × 10^5^ ± 6.3 × 10^4^ particles/m^3^
1.0 µm ≤ d_p_ < 3.0 µm	2.7 × 10^5^ ± 2.7 × 10^3^ particles/m^3^	4.1 × 10^5^ ± 5.9 × 10^4^ particles/m^3^
3.0 µm ≤ d_p_ < 5.0 µm	4.1 × 10^4^ ± 2.0 × 10^3^ particles/m^3^	8.3 × 10^4^ ± 2.1 × 10^4^ particles/m^3^
Fungal bioaerosol concentration	411 ±36 CFU/m^3^	46 ± 10 CFU/m^3^
Bacterial bioaerosol Concentration	14 ± 2 CFU/m^3^	2 ± 2 CFU/m^3^
